# Phosphorus Limitation Improved Salt Tolerance in Maize Through Tissue Mass Density Increase, Osmolytes Accumulation, and Na^+^ Uptake Inhibition

**DOI:** 10.3389/fpls.2019.00856

**Published:** 2019-07-03

**Authors:** Hongliang Tang, Le Niu, Jing Wei, Xinying Chen, Yinglong Chen

**Affiliations:** ^1^College of Life Science, Hebei University, Baoding, China; ^2^State Key Laboratory of Soil Erosion and Dryland Farming on the Loess Plateau, Northwest A&F University, Yangling, China; ^3^Institute of Soil and Water Conservation, Chinese Academy of Sciences, Yangling, China; ^4^UWA School of Agriculture and Environment, The Institute of Agriculture, The University of Western Australia, Perth, WA, Australia

**Keywords:** phosphate deficiency, root growth, salt tolerance, stress physiology, osmolytes accumulation

## Abstract

Low phosphorus (P) availability and salt stress are two major constraints for maize (*Zea mays* L.) growth in north China. A combination of salinity and high P rather than low P is more detrimental to the growth of maize. However, little is known about the mechanisms by which P nutrition modifies the salt tolerance and P uptake of maize. The present study aimed to investigate the combined effects of salinity and P on maize growth and P uptake, and to address the physiological mechanisms of salt tolerance influenced by P availability in maize. Seedlings of a local maize cultivar XY335 were grown hydroponically for 35 days under low (5 μM) or sufficient P supply (200 μM) with or without 100 mM NaCl. Root morphological traits, tissue mass density, leaf osmolytes (sugars and proline) accumulation, and Na^+^/K^+^ ratio were measured to allow evaluation of the combined effects of salinity and P on maize growth and P uptake. Both P deficiency and salinity markedly reduced the growth of maize. However, P deficiency had a more pronounced effect on shoot growth while salinity affected root growth more prominently. Combined effects of P deficiency and salinity on total root length, root surface area, and average root diameter were similar to that of plants grown under salt stress. The combination of P deficiency and salinity treatments had a more pronounced effect on tissue mass density, leaf proline and soluble sugars compared to individual treatment of either low P or NaCl. When exposed to salt stress, maize plants of sufficient P accumulated greater amount of Na^+^ than those under P deficit, but similar amounts of K^+^ were observed between the two P treatments. Salt stress significantly increased shoot P concentration of maize with sufficient P (*P* < 0.01), but not for P-deficient plants. In sum, shoots and roots of maize exhibited different responses to P deficiency and salinity, with more marked effect of P deficiency on shoots and of salinity on roots. P deficiency improved salt tolerance of maize plants, which was associated with the increase of tissue mass density, accumulation of osmolytes, reduction of Na^+^ accumulation, and selective absorption of K^+^ over Na^+^.

## Introduction

Phosphorus (P) is an essential macro-element required by plants involved in many metabolic processes including energy transfer, signal transduction, biosynthesis of macromolecules, photosynthesis, and respiration (Raghothama and Karthikeyan, 2005). Most of soil P exists in plant-unavailable forms despite the large amount of total P (Schachtman et al., 1998). Low P availability limits crop production in many low input systems of agriculture, especially in regions where calcareous and alkaline soils largely prevail (Vance et al., 2003). Plants have developed a series of adaptive strategies to cope with this constraint (Raghothama, 1999; Vance et al., 2003). These strategies include alterations in root morphology and architecture, acidification of root rhizosphere, secretion of acid phosphatases and organic acids from roots, induction of high-affinity P transport systems, changes in carbohydrate metabolism, and formation of symbiotic association with mycorrhizal fungi (Rychter and Randall, 1994; Plaxton and Carswell, 1999; Lambers et al., 2006).

Soil salinity is one of the most harmful environmental factors affecting plant growth that occurs on approximately 20% of the world’s arable lands and nearly half of irrigated lands, where crop production is severely affected by excessive salinity (Zhu, 2001). Plants exposed to high salinity often suffer from osmotic stress, toxicity of Na^+^ and Cl^−^ ions, nutritional disorders, and oxidative stress, which lead to reduction in photosynthesis and inhibition of plant growth (Hasegawa et al., 2000; Munns and Tester, 2008). Most crops are salt-sensitive or hypersensitive plants with characteristics of low resistance to salt stress (Yokoi et al., 2002). Some mechanisms that enable plants to survive under high salinity have been identified, including ion compartmentalization, osmolytes accumulation, osmotic adaptation, selective transport and uptake of ions, ion homeostasis, and leaf salt excretion (Flowers and Colmer, 2008). For example, many plants under salinity stress accumulate soluble osmolytes such as proline (Szabados and Savoure, 2010), glycine betaine (Subbarao et al., 2001), and soluble sugars (Feng et al., 2002). Proline plays an important role as osmolyte, contributes to scavenging ROS, stabilizing membrane and proteins, buffering cellular redox potential, and inducing the expression of salt stress-responsive genes (Carillo, 2018). These soluble substances are capable of mediating osmotic adjustment, protect subcellular structures, and mitigate oxidative damage caused by free radicals in response to salt stresses (Singh et al., 2015). In addition to osmolytes accumulation, plant could selectively take up K^+^ over Na^+^, alleviating adverse impacts of salt stress. Although Na^+^ has been considered as an elicitor to facilitate osmoregulation, excess Na^+^ in cytosol has toxic effects on plants by inhibiting K^+^ uptake, hence disturbing K^+^-dependent enzymatic processes and disrupting membrane integrity (Tester and Davenport, 2003). Two major Na^+^/H^+^ antiporters, NHX1 (located in vacuolar membranes) and SOS1 (located in plasma membrane), are involved in a plant’s salt tolerance by compartmentalizing Na^+^ into the vacuoles, and/or effluxing Na^+^ from cells across membranes to maintain non-toxic levels of cytosolic Na^+^ (Shi et al., 2002; Olías et al., 2009). Therefore, manipulating NHX1 and SOS1 genes in plants is an important strategy to maintain ionic homeostasis and cope with salt stress (Yamaguchi et al., 2013).

A large number of studies have been carried out to investigate the effects of salinity or P deficiency as a separate growth-limiting factor on plant growth and nutrient uptake, but the literatures on their combined effects are still limited (Hu and Schmidhalter, 2005). An example of research done in this area is by Champagnol (1979). It has also been shown that P addition to salt-affected soils improved crop growth and yield in 34 of 37 crops studied, but did not increase salt tolerance of crops (Champagnol, 1979). Munns and James (2003) found that durum wheat in saline solution was confounded by P toxicity due to increased P uptake. Recently, Abbas et al. (2018) found that low P (10 μM) is more detrimental than 100 mM NaCl (medium salinity) for shoot and root growth of both wheat cultivars irrespective of their difference in salinity tolerance under soil culture conditions. In addition to limited literature, studies on the combined effects of salinity and P deficiency are contradictory. This disagreement concentrated mainly on the following two points: (1) P supply either increases, decreases, or does not affect the salt tolerance of many crops (Champagnol, 1979); and (2) salt stress either increases, decreases, or does not affect P accumulation in plants (Grattan and Grieve, 1992). The differences in growing conditions (soil culture, sand culture, or hydroponics), plant species or genotypes, physiological developmental stages, salinity, or P supply intensity in the substrate explain this disagreement among studies (Grattan and Grieve, 1992). In most cases, P deficiency increases salt tolerance of plants and salinity decreases P concentration in plant tissue (Martinez et al., 1996; Kaya et al., 2001).

Maize (*Zea mays* L.) is an important cereal crop that is widely grown in a range of agro-ecological environments, and its growth is greatly reduced by high salinity and P deficiency (Mollier and Pellerin, 1999; de Azevedo Neto et al., 2006). In the area of calcareous soils of northern China, maize was subjected simultaneously to salinity and P deficiency. It has been shown that salinity was more detrimental to the growth of maize in combination with a high P than it was with a low P (Nieman and Clark, 1976). Under P shortage, maize exhibited a series of complex morphological, physiological, biochemical, and developmental adaptations in the roots and shoots (Calderón-Vázquez et al., 2009). It was likely that some adaptive alterations induced by P deficiency are capable of improving salt tolerance of maize under salt stress. To our knowledge, little experimental evidence exists that identifies the mechanisms by which P nutrition and salinity interact to modify the salt tolerance of maize. To explore the physiological mechanisms of salt tolerance, a hydroponic experiment was performed to obtain information on the responses of maize (*Zea mays* L. “XY335”) to the combined effects of salinity and P, and to determine how these two factors interact with one another and influence salt tolerance and P uptake of maize.

## Materials and Methods

### Plant Growth

Seeds of maize (*Zea mays* L.) cv. XY335 (a local commonly planted cultivar) were surface-sterilized in 10% (v/v) H_2_O_2_ for 15 min, washed five times in deionized water, and then germinated on moist filter paper for 5 days at 25°C in darkness. Three of similar size seedlings were transferred to each plastic pots containing 5 L of a continuously aerated nutrient solution. The nutrient solution consists of the following composition (μM): Ca(NO_3_)_2_ (2000), K_2_SO_4_ (750), MgSO_4_ (650), KCl (100), H_3_BO_3_ (1), ZnSO_4_ (1), MnSO_4_ (1), CuSO_4_ (0.1), (NH_4_)_6_Mo_7_O_24_ (0.05), and Fe-ethylene diamine tetraacetic acid (EDTA) (10). Phosphorus (P) was added to the nutrient as KH_2_PO_4_ at a concentration of 5 μM P (low P) and 200 μM P (high P). The pH of nutrient solution was adjusted to 5.8 with 0.05 M NaOH. Potassium (K) was balanced by supplying appropriate concentrations of KCl to the P-deficient treatments. After 5 days of shoot growth, 100 mM of exogenous NaCl was supplied to the nutrient solution. The nutrient solution was refreshed with an addition of 100 mM NaCl at 5-days intervals until harvest. The growth continued for 35 days. The experiment was a two-factorial (salinity and P) completely randomized design and grouped into the following four treatments: (1) high P without the addition of NaCl (CK); (2) high P with 100 mM NaCl (salt stress); (3) low P without the addition of NaCl (P deficiency); and (4) low P with 100 mM NaCl (interactive effect). There were four replicates for each treatment. The experiments were carried out in a growth chamber with a light/dark regime of 16/8 (h), temperature of 28/25 (day/night), and light intensity of 300 μmol m^−2^ s^−1^. The pots were completely randomized and re-positioned weekly to minimize the environmental effects.

### Plant Harvest and Dry Matter

**Figure 1:**
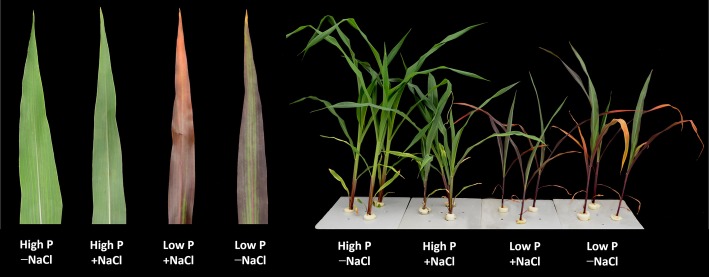
Maize plants grown for 35 days under low P (5 μM) and high P (200 μM), with or without 100 mM NaCl showing symptoms of salt stress in particular. Insert: images of representative leaves of the plants of the respective treatments.

The experiment was terminated for assessments when maize exhibited significant difference between salt treatments or P treatments after 35 days of growth (Figure 1). At harvest, roots and shoots were separated. The fresh weight (FW) was immediately determined after being washed with deionized water and blotted with filter paper. Subsamples of shoots were stored at −80°C in preparation for biochemical analysis. Roots were sealed in plastic bags and preserved at −20°C for analysis of root morphological traits. Dry weight (DW) was determined after oven-drying samples at 70°C for 72 h until constant weight was achieved. Root mass ratio (RMR, %) was calculated according to the equation:

RMR=RDW/(SDW+RDW)×100%

where SDW is shoot dry weight and RDW is root dry weight in grams.

### Root Morphology

Roots were thawed, carefully spread over a plastic box, and scanned using an Epson Perfection V750 PRO digital scanner. Data of total root length and root surface area were acquired by processing the scanned root images using the WinRHIZO image analyzing system (Regent Instructions, Quebec, Canada).

### Relative Growth Rate, Tissue Mass Density, and Tissue Water Content

The relative growth rate (RGR) was calculated using the following equation (Hoffmann and Poorter, 2002):

$$\mathrm{RGR}\;\;\left(g\;d^{-1}\right)\;\;=\;\;\left(\ln{\mathrm{DW}}_{2}\;\;-\;\;\ln{\mathrm{DW}}_{1}\right)\;/\;\left(t_{2}\;\;-\;\;t_{1}\right)$$

where *ln* is natural logarithm; DW is dry weight in g; *t* is time of harvest (d); and numbers 1 and 2 refer to first and second harvest, respectively. Tissue mass density of shoots was determined based on the method described by Ryser (1996). Specifically, tissue mass density (tissue dry biomass content) was expressed as the tissue DM/FM ratio. Tissue water content (TWC) was determined by using the following equation:

$$\mathrm{TWC}\;\;\;\left(\mathrm{ml}\;g^{-1}\;\mathrm{DW}\right)\;\;=\;\;\left(\mathrm{FW}\;\;-\;\;\mathrm{DW}\right)\;/\;\mathrm{DW}$$

### Inorganic Ion and Organic Compounds

The oven-dried samples were ground to a fine powder. Subsamples were digested in concentrated H_2_O_2_-H_2_SO_4_, and P concentration was determined using the vanado-molybdate methods as described by Westerman (1990). The concentrations of Na^+^ and K^+^ were determined using flame emission photometry (Corning, Cambridge, UK) after extraction of dry powder in 0.5% nitric acid. Proline was quantified spectrophotometrically by the ninhydrin method as described by Bates et al. (1973). Soluble sugars were quantified with the anthrone reagent according to the method as described by Yemm and Willis (1954).

### Statistics

Data were subjected to two-way analysis of variance (ANOVA) in SPSS 13.0 (SPSS Inc., USA) to examine the impacts of salinity, P, and their interactions on dry matter, relative growth rate, tissue mass density, root morphological traits, inorganic ion concentration, and organic compound concentration. Means are presented with standard error (SE). Significant difference between mean of treatments was compared using Tukey’s HSD test at the 5% probability level (*P* ≤ 0.05).

## Results

### Dry Matter Accumulation and Allocation

Two-way ANOVA showed significant effects of P, salinity, and their interaction on shoot dry weight (SDW), root dry weight (RDW), and root mass ratio (RMR), except for the effect of P on RDW (Figure 1, Table 1). Compared with high P, low P and salinity markedly reduced SDW and RDW, with a more pronounced effect of P deficiency on SDW and of salinity on RDW (Table 2). Low P and salinity caused a significantly higher RMR than individually applied high P, with a more pronounced effect of P deficiency on RMR. At high P supply, salinity significantly reduced SDW and RDW and increased RMR. Comparatively, SDW, RDW, and RMR were not significantly affected by salinity at low P supply.

**Table 1:** Two-way analysis of variance (ANOVA) on the effects of P, salinity, and their interactions on dry matter accumulation, shoot growth rate, shoot tissue density, shoot nutrient concentration, root morphological parameters, and solute accumulation in maize.

Parameters	Source of variation					
	Phosphorus		Salinity		P × Salinity	
	*F*	*p*	*F*	*p*	*F*	*p*
Shoot dry weight	250.4	<0.001	69.7	<0.001	58.3	<0.001
Root dry weight	0.06	0.813	32.5	<0.001	16.8	0.001
Root mass ratio	898.7	<0.001	6.02	0.030	4.7	0.048
Total root length	78.9	<0.001	204.7	<0.001	58.6	<0.001
Root surface area	99.9	<0.001	226.2	<0.001	87.8	<0.001
Average root diameter	19.8	0.001	81.9	<0.001	4.05	0.067
Relative growth rate	250.4	<0.001	69.7	<0.001	58.4	<0.001
Tissue mass density	113.4	<0.001	111.1	<0.001	0.11	0.748
Tissue water content	222.9	<0.001	187.2	<0.001	5.60	0.036
Shoot P concentration	1034.6	<0.001	13.7	0.003	12.6	0.004
Shoot K concentration	0.30	0.592	353.2	<0.001	24.0	<0.001
Leaf proline	144.4	<0.001	37.7	<0.001	6.62	0.024
Leaf soluble sugar	288.4	<0.001	12.3	0.004	2.47	0.142

**Table 2:** Effect of phosphorus and salinity on the biomass accumulation and allocation in maize.

P supply	Salinity	Shoot dry weight (g)	Root dry weight (g)	Root mass ratio (%)
High P	−NaCl	2.634 ± 0.15a	0.494 ± 0.03a	15.782 ± 0.10c
	+NaCl	1.322 ± 0.05b	0.306 ± 0.02c	18.794 ± 0.66b
Low P	−NaCl	0.708 ± 0.03c	0.411 ± 0.01b	36.761 ± 0.43a
	+NaCl	0.649 ± 0.02c	0.380 ± 0.01b	36.953 ± 1.04a

*The values are mean ± SE (n = 4). Different letters within each column denote significant differences (P ≤ 0.05) between treatments*.

### Root Morphological Traits

**Figure 2:**
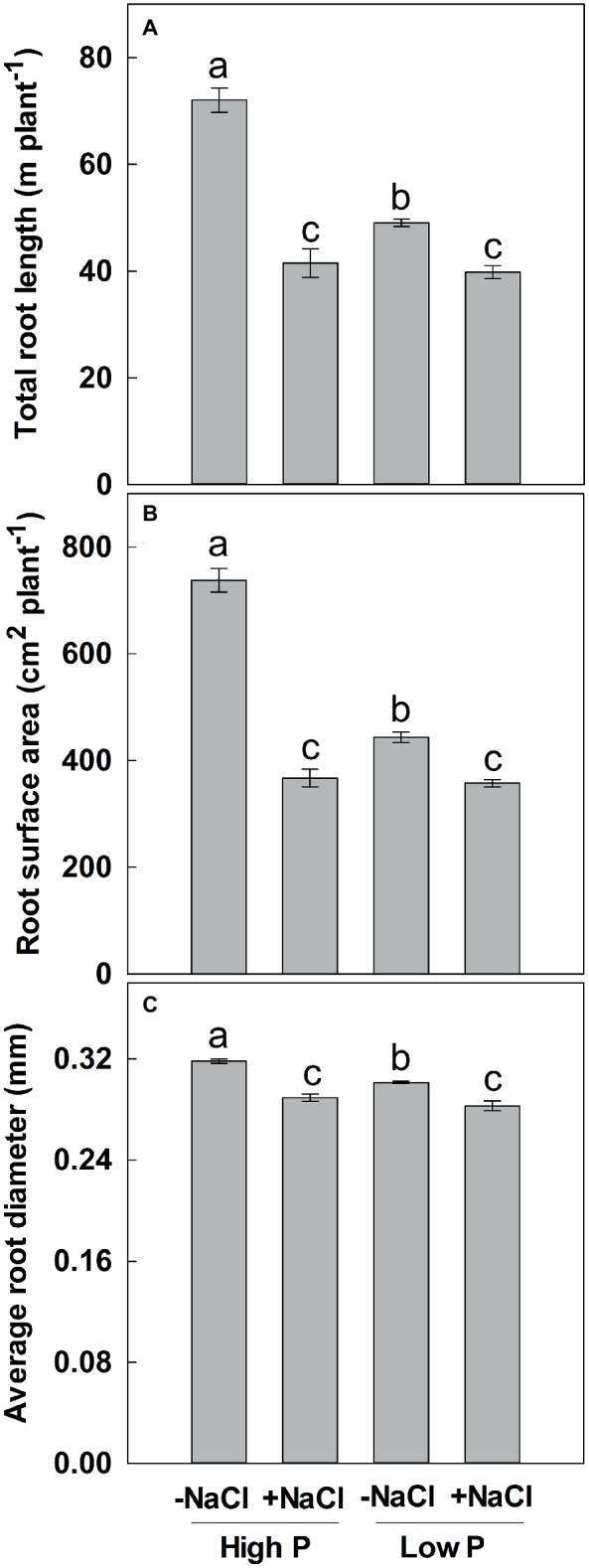
Effect of phosphorus and salinity on total root length **(A)**, root surface area **(B)**, and average root diameter **(C)**. Maize was grown for 35 days under low P (5 μM) and high P (200 μM), with or without 100 mM NaCl. Each value represents the mean (±SE) of four replicates. Different letters indicate significant differences between treatments for a given root trait (*P* ≤ 0.05).

Two-way ANOVA showed significant effects of P, salinity, and their interaction on TRL, RSA, and ARD, except for a combined effect of P and salinity on ARD (Table 1). Low P and salinity significantly decreased TRL, RSA, and ARD, with a more pronounced effect of salinity on TRL, RSA, and ARD (Figure 2). The combined effect of P deficiency and salinity on TRL, RSA, and ARD was similar to that of plants grown under salt stress. Regardless of P supply, salinity significantly reduced TRL, RSA, and ARD of maize, but a more marked reduction of TRL and RSA was observed at high P supply than at low P supply (Figure 2).

### Relative Growth Rate, Tissue Mass Density, and Tissue Water Content

**Figure 3:**
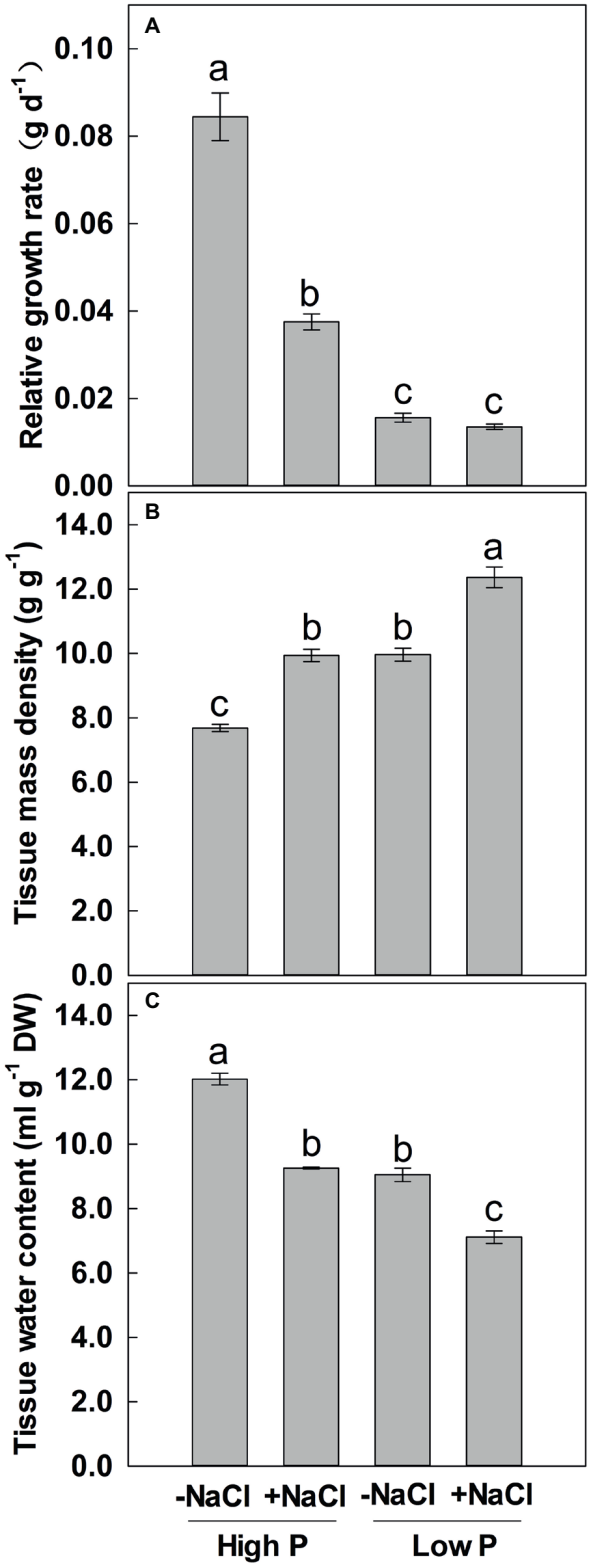
Effect of phosphorus and salinity on relative growth rate **(A)**, tissue mass density **(B)**, and tissue water content **(C)**. Maize was grown for 35 days under low P (5 μM) and high P (200 μM), with or without 100 mM NaCl. Each value represents the mean (±SE) of four replicates. Different letters indicate significant differences between treatments for a given root trait (*P* ≤ 0.05).

Two-way ANOVA showed significant effects of P and salinity on RGR, TMD, and TWC, but no significant interaction between P and salinity for TMD (Table 1). Low P and salinity significantly reduced RGR and TWC of maize, with a more marked effect of P deficiency on RGR (Figures 3A,C). A combination of P deficiency and salinity caused the lowest TWC (Figure 3C). At high P supply, RGR was significantly reduced by salinity, but not significantly affected by salinity at low P supply (Figure 3A). Low P and salinity significantly increased TMD, a combination of P deficiency and salinity caused a highest level of TMD in maize. Regardless of P supply, salinity significantly increased TMD (Figure 3B), but decreased TWC of maize (Figure 3C).

### Ion Concentration

Two-way ANOVA showed significant effects of P, salinity, and their interaction on concentration of P and K^+^ in shoots, except for effect of P on shoot K^+^ concentration (Table 1). Regardless of salinity, low P supply significantly decreased shoot P concentration. At high P supply, salinity resulted in a significant increase in shoot P concentration, which was not significantly affected by salinity at low P supply (Table 3). Shoot K^+^ concentration was significantly reduced by salinity at both P levels, but was not significantly affected by P supply with or without the salinity (Table 3). Shoot Na^+^ concentration was significantly higher in the treatments supplied with high P than ones supplied with low P with salinity, whereas Na^+^ was not detected in the treatments without salinity. Correspondingly, the ratio of K^+^ to Na^+^ was significantly lower in the treatments supplied high P than ones supplied low P with salinity (Table 3).

**Table 3:** Effect of phosphorus and salinity on the concentration of shoot P, K, Na, and K^+^/Na^+^ in maize.

P supply	Salinity	Shoot P concentration (μmol g^−1^ DW)	Shoot K^+^ concentration (μmol g^−1^ DW)	Shoot Na^+^ concentration (μmol g^−1^ DW)	K^+^/Na^+^
High P	−NaCl	222.46 ± 11.42b	1377.51 ± 16.43a	n.d.	–
	+NaCl	271.27 ± 6.94a	1015.39 ± 21.98b	486.70 ± 5.98a	2.09 ± 0.06b
Low P	−NaCl	30.01 ± 0.76c	1311.01 ± 7.32a	n.d.	–
	+NaCl	31.06 ± 1.33c	1098.73 ± 11.30b	215.31 ± 14.26b	5.17 ± 0.12a

*The values are mean ± SE (n = 4). Different letters within each column denote significant differences (P ≤ 0.05) between treatments. n.d.: not detected*.

### Soluble Sugar and Proline

**Figure 4:**
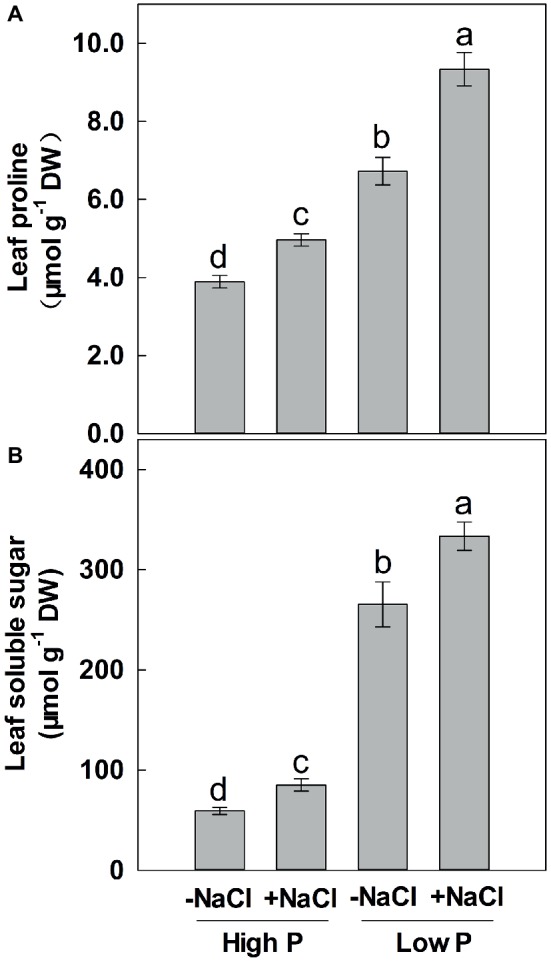
Effect of phosphorus and salinity on leaf proline **(A)** and soluble sugars **(B)**. Maize was grown for 35 days under low P (5 μM) and high P (200 μM), with or without 100 mM NaCl. Each value represents the mean (±SE) of four replicates. Different letters indicate significant differences between treatments for a given root trait (*P* ≤ 0.05).

Two-way ANOVA showed significant effects of P and salinity on the soluble sugars and proline in leaves, but no significant interaction between P and salinity for leaf soluble sugars (Table 1). Low P resulted in significant increases in soluble sugars and proline of leaves, with a more marked effect with low P as compared to high P (Figure 4). A combination of P deficiency and salinity caused the highest level of soluble sugars and proline in the leaves of maize. At both P levels, salinity caused a significant accumulation of soluble sugars and proline, but the difference was more pronounced at low P than at high P supply (Figure 4).

## Discussion

When simultaneously exposed to nutrient deficiency and salinity, plants often have a trade-off between two stresses, and their growth is controlled by the most growth-limiting factor (van der Ploeg and Kirkham, 1999). The findings of this study are in agreement with this statement, since shoot growth of maize was reduced more markedly under P deficiency than under high salinity, suggesting that P deficiency has a greater effect to maize shoot growth than salinity stress (Figure 1, Table 2). It has been frequently suggested that shoots are organs that are affected more by nutrient deficiency (e.g., deficiency of N, P, Fe) than by salinity (Rogers et al., 2003; Shenker et al., 2003; Zribi et al., 2015). When subjected to P deficiency, shoot growth was more reduced than root growth and therefore resulted in a significant increase of biomass allocation to roots (Mollier and Pellerin, 1999). In the present study, root growth of maize was reduced more markedly by salinity rather than by P deficiency (Figure 2, Table 2), suggesting that salinity plays a predominant role in determining root growth. This result is consistent with previous studies in *Lycopersicon esculentum* (Mohammad et al., 1998), *Hordeum vulgare* (Zribi et al., 2011), and *Aeluropus littoralis* (Zribi et al., 2015). Taken together, our results suggested that the growth of shoots and roots exhibited different responses to P shortage and salinity, with a more pronounced impact of P deficiency on shoots and of salinity on roots.

It has been known that P availability can regulate the salt tolerance of plants (Champagnol, 1979; Rogers et al., 2003; Phang et al., 2009; Zribi et al., 2011, 2012, 2015). In most of cases, P-deficient plants exhibited an improved salt tolerance level as compared with P-sufficient plants. In the present study, salinity did not significantly affect the shoot growth of maize under low P supply, while it significantly reduced shoot growth of maize with sufficient P supply, suggesting that P-deficient maize plants are more salt tolerant than P-sufficient plants. These results are consistent with previous reports by Phang et al. (2009) in soybean and Zribi et al. (2011) in barley. It is likely that some alterations induced by P deficiency may partly be capable of explaining the greater salt tolerance of P-deficient plants.

In this study, P-deficient plants exhibited a lower RGR and higher TMD as compared with P-sufficient plants (Figures 3A,B), which is consistent with the result of Louw-Gaume et al. (2010). Rapid growth is required for rapid resource acquisition, which is achieved through rapid expansion of low-density tissue (Ryser and Lambers, 1995). However, a slower growth together with higher tissue mass density is typical for plants from low productive environments (Poorter and De Jong, 1999). It is therefore considered that rapid growth is more responsive while high-density tissue is more resistant to environment stresses. Shoot growth of P-sufficient plants was greatly reduced under salt stress as fast growth with low-density tissue, but that of P-deficient plants was not significantly affected by salinity as slow growth with high-density tissue, indicating that salt tolerance was associated with growth rate and tissue mass density. In addition, nutrient ion content in leaves might be correlated with growth inhibition under salinity. Neves-Piestun and Bernstein (2005) suggested reduced levels of Ca and K content, and toxic levels of Fe as possible causes of growth inhibition in maize leaves, and Na and Cl accumulation were not correlated with growth inhibition under salinity. In present study, salt stress caused a significant decrease of shoot K concentration in P-deficient and P-sufficient maize while growth was significantly unaffected by salinity for P-deficient maize, suggesting that reduction of shoot K^+^ concentration is correlated with inhibition of maize growth. Further, shoot Na^+^ concentration is much higher under P sufficiency than under P deficiency when maize was exposed to 100 mM NaCl, indicating that salinity plays a minor role in shoot growth inhibition of maize.

Previous studies have suggested that P deficiency reduced root hydraulic conductance and whole plant water potential, probably by lowering the activity of water channel protein (Radin and Eidenbock, 1984; Clarkson et al., 2000; Li et al., 2009). Our results support this statement, since we found that TWC was significantly reduced under P deficiency. Zribi et al. (2011) indicated that leaf water content was correlated with leaf osmotic potential under P deficiency. When exposed to salinity, a further reduction in TWC was observed at both low and high P supply. This trend was associated with a high accumulation of two major compatible solutes, proline and soluble sugars, which usually reduce osmotic potential of plants. The soluble sugars usually accumulate in P-deficient plants, since utilization of the photoassimilate was largely restricted (Gibson, 1988; Wissuwa et al., 2005). However, some studies suggested that salinity has no significant effect on soluble sugars accumulation (Zribi et al., 2011; Moinuddin et al., 2014). Comparatively, proline seems to be largely induced by salinity instead of P deficiency (Zribi et al., 2012, 2015). These results were not in agreement with this study, since we found that soluble sugars and proline were induced simultaneously by P deficiency and salinity (Figure 4). More importantly, we have indicated that soluble sugars and proline were more accumulated in P-deficient maize plants under salinity than those exposed to combination of salinity and sufficient P supply, suggesting that soluble sugars and proline were involved in P deficiency-induced osmotic adjustment for salt tolerance.

Ferchichi et al. (2018) indicated that wild and cultivated *Hordeum* genotypes with contrasting salt tolerance display different patterns of metabolites under 3 week 200 mM NaCl treatment, suggesting that the tolerance of plants to salinity is largely dependent on the duration of stress. In the early stage of stress, *H. maritimum* plants were able to maintain lower leaf concentrations of sodium and chloride, and higher concentrations of potassium compared with *H. vulgare*. Over prolonged stress, accumulation of proline, together with glycine betaine to osmotically balance *H. maritimum* leaves, was in concomitance with the decrease of potassium-to-sodium ratio, the increase of hydrogen peroxide, and decrease of chlorophylls. In our study, we examine these soluble osmolytes in shoots, but not in roots at single stage, which might result in an incomplete conclusion. It has been considered that leaf is the final reflection for the intensity of stress, and thus changes in leaf soluble osmolytes (such as soluble sugar and proline) and salt content could well reflect the responses of maize to salinity and P shortage. Leaf osmotic substance concentration is commonly used as an evaluation of plants’ response to different stresses. A recent study observed alleviated effects to salt stress by applying exogenous methyl jasmonate (0.03 mM), a volatile organic compound used in plant defense and some developmental pathway, in the recretohalophyte *Limonium bicolor* (Yuan et al., 2018). Whether methyl jasmonate played an important role in improving plants’ tolerance to salinity stress in maize under low P condition requires further investigation.

The Na^+^ in tissues is usually involved in salt tolerance of plants due to its toxic effect and oxidative damage on plants (Greenway and Munns, 1980). These adverse effects may be alleviated by reducing Na^+^ concentration and maintaining a higher cytosolic K^+^/Na^+^ ratio under salt stress (Blumwald, 2000). One key trait of salt tolerance is the ability of plants to reduce Na^+^ concentration. Cuin et al. (2009) suggested that Na^+^ exclusion from the shoot correlated with salinity tolerance in bread wheat (*Triticum aestivum* L.) and durum wheat (*Triticum turgidum* L. ssp. durum) treated with 150 mM NaCl. In the present study, low P supply significantly decreased the concentration of Na^+^ in shoots under salt stress and thus improved salt tolerance of maize (Table 3). This result was consistent with the previous results reported by Phang et al. (2009) in soybean and Zribi et al. (2011) in barley. When exposed to NaCl, roots are important for countering injuries from salinity by sequestering Na^+^ in the vacuole to improve salt tolerance. Annunziata et al. (2017) suggested that 100 mM of NaCl leads to an increase of sap osmolality from about 305 mOsmol kg^−1^ in control roots to about 530 mOsmol kg^−1^ in roots under salinity in durum wheat. Root cells of wheat sequester sodium in the vacuole to adapt salinity as a cheap osmoticum for osmotic adjustment. It is possible that maize may accumulate higher Na^+^ in roots under low P than under high P when plants were exposed to 100 mM NaCl, which prevents Na^+^ from entering into shoots. Another key trait of salt tolerance is the ability of plants to selectively accumulate K^+^ over Na^+^, resulting in a higher K^+^/Na^+^ ratio (Chen et al., 2007). Cuin et al. (2009) indicated that shoot sap K^+^, rather than total shoot K^+^ content, is needed to provide efficient osmotic adjustment under saline conditions. In contrast to barley, NaCl-induced K^+^ efflux from seedling roots did not predict salinity tolerance in wheat, implying that shoot, not root K^+^ retention, is important for wheat, which is in contrary with barley as shown in Chen et al. (2007). Although we did not observe a alteration in shoot K^+^ concentration between two P levels under salt stress (Table 3), a higher K^+^/Na^+^ ratio was obtained since Na^+^ concentration was lower in the treatment with a combination of P deficiency and salinity than that of P sufficiency and salinity. These results suggested that Na^+^ concentration and K^+^/Na^+^ ratio in shoots were associated with salt tolerance in maize. It has been suggested that changes in root morphological and physiological traits contribute to the uptake of nutrient ion (Grattan and Grieve, 1992). Therefore, the change in shoot Na^+^ concentration was closely related to the changes in these root traits. It is unlikely that root morphological traits contributed to the increase in shoot Na^+^ concentration under a combination of salinity and sufficient P supply, since TRL, RSA, and ARD did not exhibit a significant difference between low P and high P under salt stress (Figure 2). As a result, increase in shoot Na^+^ concentration was naturally attributed to the changes in root physiological uptake. Rubio et al. (2005) found a sodium-dependent high-affinity P transporter at the plasma membrane from the leaf and root cells of *Zostera marina*, indicating that a synergistic effect may exist between Na^+^ and P uptake. Consistent with the findings, we showed that exogenous high P improved Na^+^ concentration in shoots while low P reduced it under salt stress, suggesting that Na^+^ uptake was improved by exogenous P supply. Besides the contribution of root morphological traits, extrusion of Na^+^ is an important mechanism to maintain a relatively low Na^+^ concentration and high K^+^/Na^+^ ratio under saline conditions, thus conferring tolerance to salt stress. As a putative Na^+^/H^+^ antiporter in the plasma membrane, SOS1 (salt overly sensitive) mediated Na^+^ extrusion from cytoplasm and controlled long-distance Na^+^ transport in *Arabidopsis* (Shi et al., 2002). A higher level of expression of SOS1 in *Medicago falcata* accounts for the greater tolerance to salt stress conditions (Liu et al., 2015). Although no evidence was presented here, it can be inferred that reduction of Na^+^ concentration in P-deficient maize plants under salt stress was associated with high expression of SOS1, causing a high tolerance to salt stress.

It is understandable that experimental findings of plant response to salt stress are variable (Shavrukov, 2013). Apart from crop species being studied, how salt applied to the plants, gradually or suddenly, also influences the interpretation of the results (Almansouri et al., 1999; Shavrukov, 2013; Woodrow et al., 2017). The present study exposed the maize plants to 100 mM NaCl and progressive imposition method should be used to avoid salt shock in the further studies on salinity.

## Data Availability

All datasets generated for this study are included in the manuscript and/or the supplementary files.

## Author Contributions

HT and YC designed the experiment. LN, JW, and XC conducted the experiment and collected data for preliminary analysis. HT and YC further analyzed the data and prepared the manuscript. All authors reviewed and commented on the manuscript. HT and YC revised the manuscript.

### Conflict of Interest Statement

The authors declare that the research was conducted in the absence of any commercial or financial relationships that could be construed as a potential conflict of interest.
